# The Royal Institute of Public Health: Plymouth Congress

**Published:** 1922-07

**Authors:** 


### THE ROYAL INSTITUTE OF PUBLIC HEALTH: PLYMOUTH CONGRESS.

THE Royal Institute of Public Health held its annual congress at Plymouth from May 31 to June 5. The subjects discussed were divided into four sections : " State Medicine and Municipal Hygiene," " Naval, Military and Air," " Bacteriology and Bio-chemistry," and " Women and Public Health."

The newly-elected president, Lord Fortescue, paid a tribute to the work of nursing associations. One of the great needs of the nation was the raising of the general standard of health and physique. There was a doubt whether all their energy and expenditure had been wisely directed, and whether, instead of improving the general average health of their people, they had not in some respects lowered it by keeping alive sickly babies and ricketty youths and young women, clever often, but physically deplorable, who, instead of dying as in the old days before they could do any harm, now, thanks to the fatherly care of the State, lived just long enough to marry and to beget offspring full of hereditary taints and weaknesses. If they accepted the rejections during recruiting for 1919 and 1920, he could not see how they could avoid the conclusion that a large proportion of their male population fell far short of the standard of soundness proper to their age, and even if the female half was in much better case there seemed little prospect under present conditions of a larger proportion of the next generation being more capable than the last of passing the doctor. He felt that the housing question was the most urgent of all. If there were decent dwellings for all, scores of difficulties in regard to disease would disappear. If our population were " C 3," could we hope to fill up or hold our " A 1 " Empire ? The two problems of filling up and holding were allied. Empty countries were a temptation to other people, and a sparse white population ran risks of its own if vastly outnumbered by coloured peoples. Both Australia and South Africa needed filling up badly, but they had no use for men who were unsound. Nor could we afford to send away too many of our best, for the proportion of the physically infirm was already too large here. Our people had begun to appreciate, as they did not a generation ago, the greatness of our heritage and their own duty to it. Those engaged in public health work would do great service if, by reducing the number of the " C 3 " class, they could make the people of the Mother Country better fitted physically for the responsibilities of the Empire.

Dr. T. Stenhouse Williams, of the Dairy Research Institute, Reading, speaking on " How to Improve the Milk Supply," said that legislation had effected nothing, or very little, in the improvement of milk. His impression was that a product which would never go sour in the home would cost 1d. a quart more. Merely to say they would have Grade A milk was not enough. The wisest thing was to urge upon the Ministry of Agriculture to set up in every agricultural college a sufficient staff of men prepared to watch over the milk of the Grade A farmer in their district from its production to its distribution. He felt there were grave objections to setting up legislative Pasteurisation institutes in the country.

Mr. A. T. Loram (Exeter) thought it was not by legislation but by sympathetic help given to the farmer that they would get a cleaner milk supply. The public would give 1s. a quart for beer, but few would be willing to pay 1s. a quart for milk, and among them the doctors were the fewest. Members of the profession were found as governors of hospitals, but it was the lowest milk tender that was accepted, without much regard to any guarantee of its quality. A greater danger to public health than water adulteration was contamination.

Mr. Wilfred Buckley, of the Pure Milk Society, expressed surprise that people associated with child welfare work did not more actively engage in a campaign to secure a more wholesome milk supply. Classification of milk into grades, although it might tend to the production of grade-milk, where the poor would suffer, would lead in the long run to the education of the farmer and an improvement in the milk supply all round.

Sir Thomas Oliver, dealing with "Some of the Social and Medical Gains of Industrial Legislation," said that for fair play, justice and protection our country was an example for others to follow. Its trend had been to give to children more opportunity of home life, fuller play, and a varied education. Motherhood in the case of the poorer working classes had been shorn of some of the anxiety of previous years. Welfare work sought to promote a better understanding between employer and employed, based upon just dealing and mutual co-operation. Among the medical gains secured by industrial legislation were the marked decline of lead poisoning and the total disappearance of phosphorus poisoning in the manufacture of lucifer matches.

In the Naval and Military Section, Major F. W. Dawson said the most dangerous carrier of disease was the common cow. In New Zealand, a country filled with enthusiasm for the care of the young, considerable progress was being made in the campaign against tuberculosis in cattle. Each year the incidence of tuberculosis in the country was less.

Dealing with war wastage through minor ailments, Colonel A. B. Soltau spoke of the importance of diet to the army in the field, and its effect in regard to skin diseases. He referred to the deficiency of vegetables and the destruction of vitamines by overcooking in Army stew as one of the causes of pyodermia.

In the course of a discussion on venereal disease, Dr. Kenneth Walker expressed the hope that differences on the subject of prophylactics were being settled. He was of opinion that clinics had failed, although they were costing an immense amount of money, and the number of patients who broke off treatment while still in a condition of infection was tremendous. The policy of persuasion having been unsuccessful, some form of compulsion should be considered. The only real argument against notification was the question of sex discrimination.

Dr. G. D. Kettlewell (Plymouth) advocated that clinics should be opened in the evening, which would be convenient to the working classes. It was because the officer in charge of the clinic had no time to talk to the patients that only 10 per cent. of them went through the whole course of treatment.

Miss Nora March said, although she favoured notification, many women's societies were against it.

Surgeon Rear-Admiral Sir Daniel McNabb said that if the same principles operating in training and maintaining health in the Services had been applied to the civil population, even in a small degree, for the generation before the Great War, they would never have been faced with an army of defectives. In the matter of recreation it was most important that something should be done for the youths of the country. They had some fine athletes, no doubt, but too many onlookers.

A national movement for the prevention of dental caries was suggested by Dr. James Wheatley (Shropshire), who gave a list of three rules: No liquid at meals; no meal to be finished with jam, marmalade or milk; no milk, biscuits, or sweets just before bedtime. After two years infants should be fed on solid foods and taught to drink only after meals. He had found children with good teeth in homes where a tooth-brush had never been seen. He would prefer that a child should be fed on hard foods and drink after meals and never use a tooth-brush.

